# P-312. Elimination of barrier contact precautions for Vancomycin-Resistant *Enterococcus* (VRE)-colonized patients was not associated with the acquisition of VRE in a tertiary care center

**DOI:** 10.1093/ofid/ofae631.515

**Published:** 2025-01-29

**Authors:** Rishi Chanderraj, Ian Kidder, Carey Dombecki, Anastasia Wasylyshyn, Laraine Washer, John Mills, Aaron King, Robert Woods

**Affiliations:** University of Michigan, Ann Arbor, MI; University of Michigan, Ann Arbor, MI; University of Michigan, Ann Arbor, MI; University of Michigan, Ann Arbor, MI; University of MIchigan, Ann Arbor, Michigan; Rutgers Robert Wood Johnson Medical School, New Brunswick, New Jersey; University of Michigan, Ann Arbor, MI; University of Michigan, Ann Arbor, MI

## Abstract

**Background:**

In April 2020, the University of Michigan discontinued barrier contact precautions for Vancomycin-Resistant Enterococcus (VRE) colonized patients while maintaining VRE surveillance. We conducted a retrospective cohort study to assess the impact of this policy change on the risk of VRE acquisition

Inclusion and Exclusion Criteria of the Cohort

**Figure:**
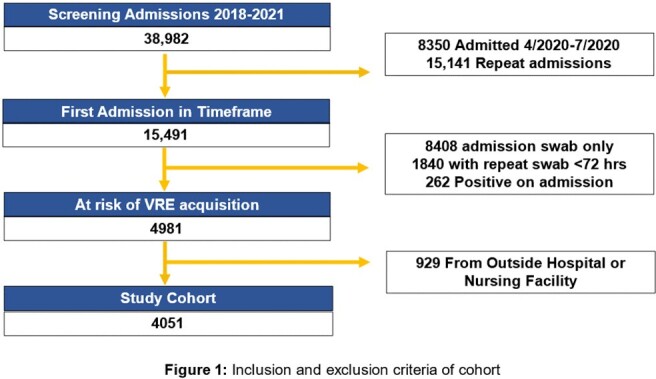

**Methods:**

We analyzed patients admitted between 1/2018 and 3/2021 who underwent weekly surveillance. The primary outcome was VRE-free survival; the main exposure was admission before or after the contact precaution policy. Demographics, Elixhauser comorbidity index, antibiotic and PPI exposure, and colonization pressure were incorporated as covariates in an extended Cox proportional hazard model of VRE-free survival. Time-varying covariates were accounted for and modeled using log-transformed time. Patients were censored at discharge or death.

VRE-free survival before and after change in contact precautions policy

**Figure:**
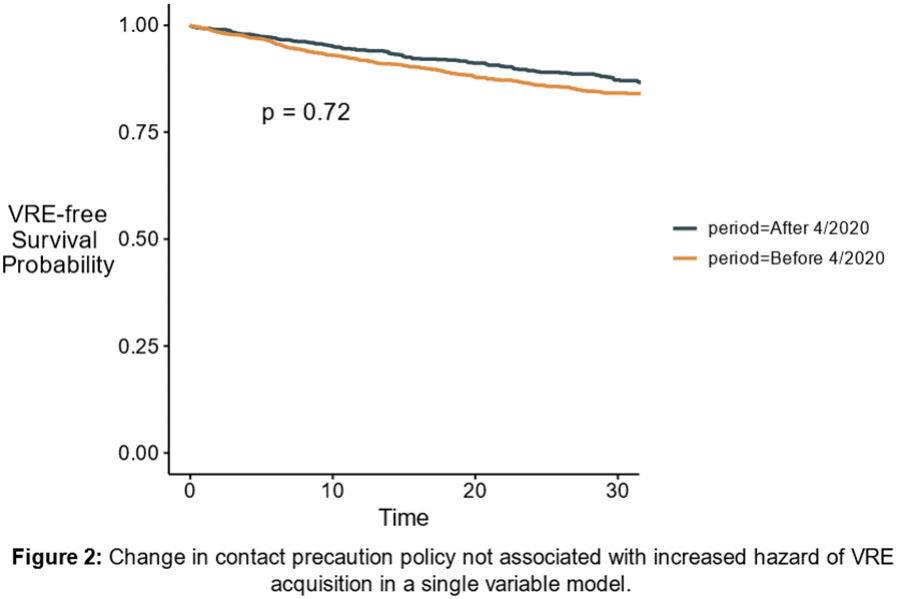

Change in contact precaution policy was not associated with an increased hazard of VRE-acquisition in single variable analysis

**Results:**

We identified 2,221 patients admitted before the policy change and 1,830 after, with 225 cases of VRE acquisition before and 196 cases after. No significant differences in age, demographics, antibiotic use, or PPI exposure were observed among VRE patients admitted before and after the policy change. However, VRE patients admitted before the policy change had more medical comorbidities (mean Elixhauser: 23.64 before, 20.77 after; p=0.008) and lower exposure to colonization pressure (mean VRE patient days: 13.4 before, 29.9 after; p=0.019).

In a multivariable extended Cox proportional hazard model, admission before or after the change in contact precaution policy showed no significant association with VRE-free survival (HR 0.81-1.1), or infection with VRE (HR 1.01, 95% CI 0.92-1.21). The most significant risk factors for VRE acquisition were vancomycin treatment (HR 1.60, 95% CI 1.25-2.03, p< 0.001) and PPI treatment (HR 1.43, 95% CI 1.15-1.77, p=0.001).

Multivariable cox-regression model of risk factors for VRE acquisition

**Figure:**
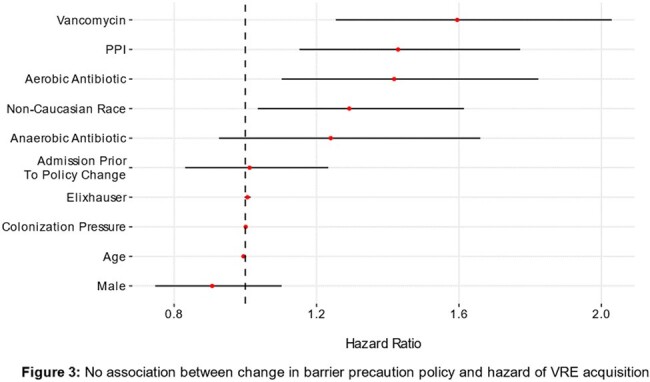

There was no association between the change in contact precautions policy and risk of VRE acquisition

**Conclusion:**

The policy change to eliminate isolation and barrier precaution requirements for VRE patients was associated with no detectible increase in the hazard of VRE acquisition. The most significant risk factors for VRE acquisition were vancomycin and PPI treatment. Our results suggest that appropriate use of PPI and antimicrobial stewardship may have a greater impact on preventing the spread and acquisition of VRE than barrier precautions.

**Disclosures:**

**Laraine Washer, MD**, Ferring Pharmaceuticals: Advisor/Consultant

